# Prognostic value of left atrial reservoir function in patients with severe primary mitral regurgitation undergoing mitral valve repair

**DOI:** 10.1093/ehjci/jeac058

**Published:** 2022-03-18

**Authors:** Jan Stassen, Aniek L van Wijngaarden, Steele C Butcher, Meindert Palmen, Lieven Herbots, Jeroen J Bax, Victoria Delgado, Nina Ajmone Marsan

**Affiliations:** Department of Cardiology, Leiden University Medical Center, Albinusdreef 2, 2300 RC, Leiden, The Netherlands; Department of Cardiology, Jessa Hospital Hasselt, Stadsomvaart 11, 3500 Hasselt, Belgium; Department of Cardiology, Leiden University Medical Center, Albinusdreef 2, 2300 RC, Leiden, The Netherlands; Department of Cardiology, Leiden University Medical Center, Albinusdreef 2, 2300 RC, Leiden, The Netherlands; Department of Cardiology, Royal Perth Hospital, 197 Wellington St, Perth WA 6000, Australia; Department of Thoracic Surgery, Leiden University Medical Center, Albinusdreef 2, 2300 RC, Leiden, The Netherlands; Department of Cardiology, Jessa Hospital Hasselt, Stadsomvaart 11, 3500 Hasselt, Belgium; Department of Cardiology, Leiden University Medical Center, Albinusdreef 2, 2300 RC, Leiden, The Netherlands; Department of Cardiology, Turku Heart Center, University of Turku and Turku University Hospital, Kiinamyllynkatu 4-8, FI-20520, Turku, Finland; Department of Cardiology, Leiden University Medical Center, Albinusdreef 2, 2300 RC, Leiden, The Netherlands; Department of Cardiology, Leiden University Medical Center, Albinusdreef 2, 2300 RC, Leiden, The Netherlands

**Keywords:** primary mitral regurgitation, mitral valve surgery, left atrial reservoir strain, prognosis

## Abstract

**Aims:**

Mitral regurgitation (MR) has a significant haemodynamic impact on the left atrium. Assessment of left atrial reservoir strain (LARS) may have important prognostic implications, incremental to left atrial (LA) volume, and conventional parameters of left ventricular (LV) structure and function. This study investigated whether preoperative assessment of LARS by speckle tracking echocardiography is associated with long-term outcomes in patients undergoing mitral valve repair for severe primary MR.

**Methods and results:**

Echocardiography was performed prior to mitral valve surgery in 566 patients (age 64 ± 12years, 66% men) with severe primary MR. The study population was subdivided based on a LARS value of 22%, using a spline curve analysis. The primary endpoint was all-cause mortality. During a median follow-up of 7 (4–12) years, 129 (22.8%) patients died. Patients with LARS ≤22% showed significantly higher mortality rates at 1-, 3-, and 5-year follow-up (6%, 12%, and 15%, respectively) when compared with patients with LARS >22% (2%, 3% and 5%, respectively, *P* < 0.001). Age [hazard ratio (HR): 1.06; 95% confidence interval (CI): 1.03–1.09; *P* < 0.001], LV global longitudinal strain (HR: 0.92; 95% CI: 0.87–0.98; *P* = 0.014), and LARS (HR: 0.96; 95% CI: 0.93–0.99; *P* = 0.014) were independently associated with all-cause mortality.

**Conclusion:**

Preoperative LARS is independently associated with all-cause mortality in patients undergoing mitral valve repair for primary MR and provides incremental prognostic value over LA volume. LARS might be helpful to guide timing of mitral valve surgery in patients with severe primary MR.

## Graphical Abstract

**Graphical Abstract:**
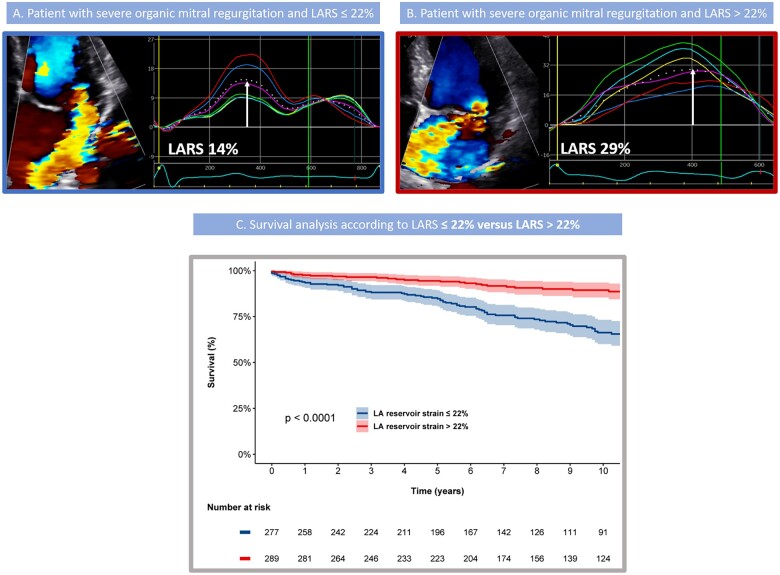
Assessment of left atrial reservoir strain and long-term survival in patients with severe primary mitral regurgitation undergoing mitral valve repair.

## Introduction

Severe primary mitral regurgitation (MR) is a growing public health problem and, when left untreated, is associated with increased morbidity and mortality.^1,2^ Prognosis in these patients can be significantly improved by mitral valve surgery^3,4^ with current guidelines recommending surgery for symptomatic patients or asymptomatic patients when left ventricular (LV) systolic dysfunction or dilatation occurs.^5,6^ Furthermore, because advances in mitral valve surgery have led to excellent long-term outcomes in experienced centres, guidelines also recommend surgical repair in asymptomatic patients when there is a high chance of durable surgical repair.^5,6^ However, early recognition of indicators of poor prognosis in these patients remains challenging, despite being essential for optimal risk stratification and timely referral for intervention. Since MR-associated cardiac remodelling affects not only the left ventricle but also the left atrium, identifying early signs of left atrial (LA) remodelling might be of clinical importance, especially considering that changes in LA size and function may occur before LV dysfunction occurs.^7,8^ Current European guidelines already suggest the consideration of mitral valve repair in the presence of significant LA dilatation [i.e. LA volume index (LAVi) ≥60 mL/m^2^] or with new onset atrial fibrillation,^6^ proposing an additional role of the left atrium in further risk stratification. However, evidence for these recommendations remains limited^9–11^ and are not included in the latest updated American guidelines,^5^ emphasizing the need for further research on the prognostic role of LA remodelling in primary MR. In this regard, assessment of LA function, rather than size, might have incremental value for further risk stratification. LA reservoir function more closely reflects LA compliance, and a reduced LA compliance may favour the development of pulmonary congestion and hypertension, and the onset of symptoms at an early stage.^12–14^ Although few studies have shown the relationship between LA function and clinical indications for mitral valve surgery, study populations were small and outcome data were lacking.^8,15^ Accordingly, the aim of the present study was to evaluate the association between LA function, assessed by speckle tracking echocardiography, and long-term outcome in a large cohort of patients with severe primary MR undergoing mitral valve repair.

## Methods

### Patient population

Patients who underwent mitral valve repair for moderate to severe and severe primary MR at the Leiden University Medical Centre, The Netherlands, between 2000 and 2019 were identified. Patients with rheumatic valve disease, active endocarditis, connective tissue disorders, hypertrophic cardiomyopathy, congenital heart disease, previous surgery, significant mitral stenosis (defined as mean gradient >5 mmHg), or significant (i.e. more than mild) aortic valve disease were excluded. Patients in whom transthoracic echocardiography before surgery was not available for analysis were also excluded. All patients included underwent complete clinical and echocardiographic evaluation before mitral valve surgery. The mean delay between the echocardiographic examination and mitral valve surgery was 1 (0–4) month. Patient information was prospectively collected in the departmental cardiology information system (EPD-vision; Leiden University Medical Centre, Leiden, The Netherlands) and retrospectively analysed. Clinical data included demographic characteristics, cardiovascular risk factors, New York Heart Association (NYHA) functional class, and comorbidities. The surgical technique for mitral valve repair has been previously described by our study group.^16^ Repair techniques used included chordal replacement for anterior mitral valve leaflet prolapse. Commissural prolapse was treated predominantly by papillary muscle head repositioning. For the posterior mitral valve leaflet, a combination of resection and neochord techniques was used. In all cases, a ring annuloplasty without downsizing was performed to stabilize the annulus and the suture line. The study complies with the Declaration of Helsinki and was approved by the Institutional Review Board. Due to the retrospective design of this study, the Medical Ethical Committee waived the need of written informed consent.

### Echocardiography

Standard transthoracic echocardiography was performed with commercially available ultrasound machines (Vivid 7 and E9, GE-Vingmed, Milwaukee, WI, USA). Electrocardiogram-triggered echocardiographic data were stored digitally in a cine-loop format for offline analysis using EchoPAC version 113 and 203 (GE Medical Systems, Horten, Norway). LV end-diastolic diameter and LV end-systolic diameter were measured from the parasternal long-axis view. LV volumes, LV ejection fraction (LVEF), and LA volumes were measured using Simpson’s biplane method and indexed for body surface area.^17^ Using tissue Doppler imaging of the mitral annulus on the apical four-chamber view, the *e*′ was measured at both the lateral and septal side, and averaged to calculate the *E*/*e*′ ratio.^17^ MR severity was quantitatively assessed according to current recommendations using a multi-parametric approach, including the effective regurgitant orifice area (using the proximal isovelocity surface area method) and regurgitant volume measurements, when feasible.^18^ Systolic pulmonary artery pressure was estimated by measuring maximal tricuspid regurgitant jet velocity with the simplified Bernoulli equation in combination with an estimation of the right atrial pressure, as recommended.^19^ Speckle tracking analysis was performed from the apical views (two-, three-, and four chambers) at a frame rate >40 fps (mean 60 fps) to assess LV global longitudinal strain (GLS).^20^ The region of interest was automatically created and manually adjusted to the myocardial thickness when necessary. LV GLS was then calculated by averaging the peak longitudinal strain values of the 17 segments, excluding segments that could not be traced correctly, and was reported as an absolute (i.e. positive) value. LA strain was measured on the apical four-chamber view, according to current guidelines.^21^ A region of interest was manually drawn along the LA endocardial border when the left atrium was at its minimum volume after atrial contraction. Automatic tracking of the LA wall by the software was visually verified and corrected by adjusting the region of interest or the width of the contour, ensuring appropriate capture of LA motion. The resulting LA strain curve provided two peaks with the first peak just before mitral valve opening representing LA reservoir strain (LARS). The average LA longitudinal strain curve was used to determine this value. LARS was chosen over LA conduit strain and LA contractile strain because it showed a good correlation with LA wall fibrosis on delayed enhancement magnetic resonance imaging,^22^ reflecting therefore atrial compliance, and is still measurable in patients having atrial fibrillation.

### Follow-up and outcome

Patients were followed-up for the primary endpoint of all-cause mortality after surgery. Data on mortality were obtained from the departmental cardiology information system (EPD-Vision, Leiden University Medical Centre, Leiden, The Netherlands), which is linked to the governmental death registry database. Follow-up data were complete for all patients.

### Statistical analysis

**Figure 1:**
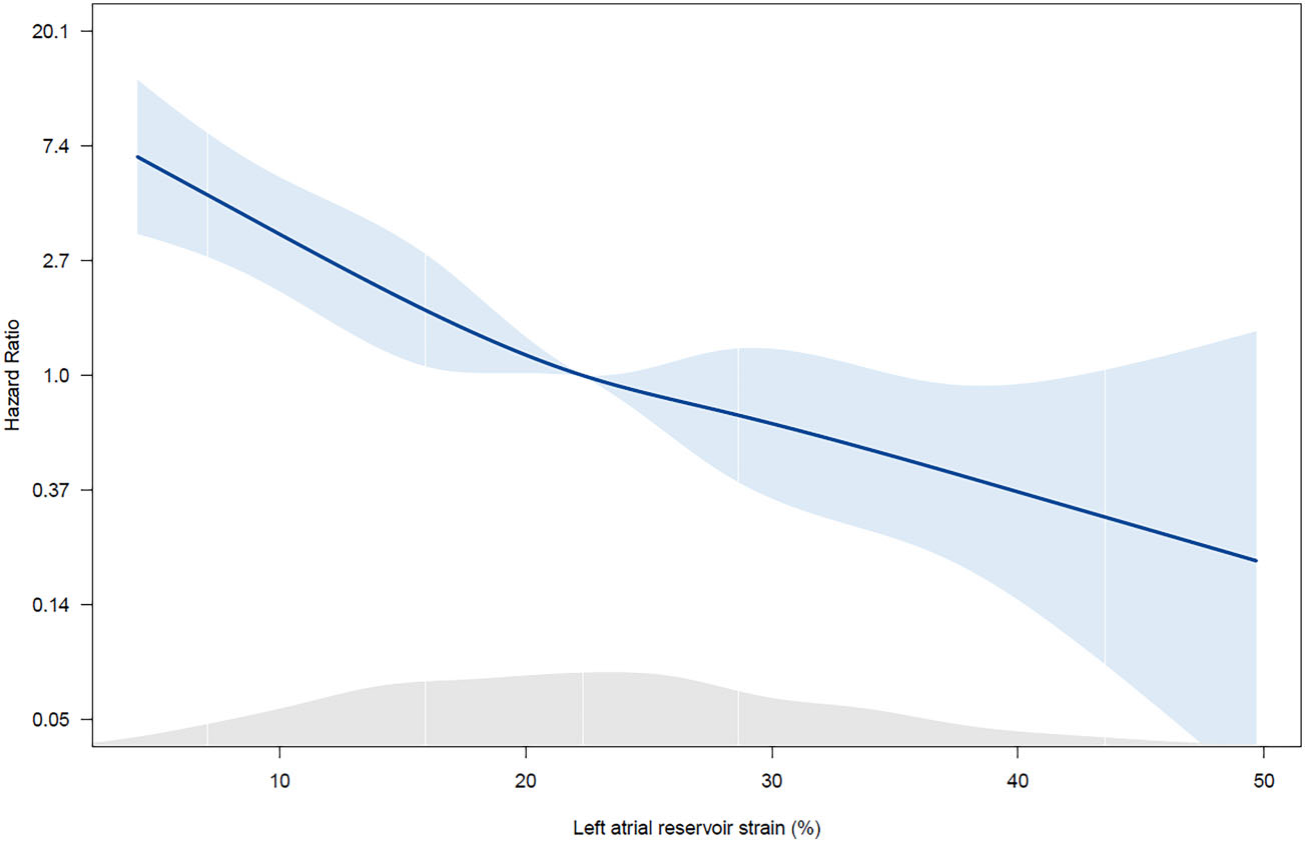
Spline curve demonstrating the hazard ratio for the occurrence of all-cause mortality at follow-up according to left atrial reservoir strain (LARS). The curve shows the hazard ratio change for the occurrence of all-cause mortality with 95% confidence intervals across a range of values of LARS at the time of the index echocardiogram. The density plot below shows the distribution of the study population according to values of LARS. A threshold of LARS to dichotomize the population can be derived from this curve (i.e. in which the predicted HR is ≥1).

Continuous variables are reported as mean ± standard deviation when normally distributed and as median (interquartile range) when not normally distributed. Categorical variables are presented as absolute numbers and percentages. Continuous variables were compared using the independent sample Student’s *t*-test when normally distributed whereas the Mann–Whitney *U*-test was used to compare continuous variables that did not adhere to a normal distribution. Categorical variables were compared using the Fisher’s exact test. Changes in hazard ratio (HR) for all-cause mortality across the LARS values (as a continuous variable) were investigated by fitting a spline curve and a threshold of 22% to dichotomize the population was derived (i.e. in which the predicted HR was ≥1, *Figure 1*). Furthermore, patients were divided into four groups according to the presence of LA dilatation and LA dysfunction based on this cut-off value for LARS and on a cut-off value of 60 mL/m^2^ for LAVi (based on current guideline recommendations^6^: Group 1—LAVi <60 mL/m^2^ and LARS >22%, Group 2—LAVi ≥60 mL/m^2^ and LARS >22%, Group 3—LAVi <60 mL/m^2^ and LARS ≤22%, or Group 4—LAVi ≥60 mL/m^2^ and LARS ≤22%). Cumulative survival rates were estimated by the Kaplan–Meier method for all-cause mortality, and a log-rank test was used to compare groups. Cox proportional hazard regression analysis was performed to investigate the association between clinical and echocardiographic parameters with all-cause mortality. The HR and 95% confidence interval (CI) were calculated and reported. In the univariable analysis, variables with a *P*-value <0.05 were considered statistically significant and entered in the multivariable model. The proportional hazards assumption was verified through the evaluation of Schoenfeld residuals. To inspect for multi-collinearity, the Pearson correlation coefficient was calculated between continuous variables, assuming no significant multi-collinearity when the correlation coefficient was <50%. In addition, the Variation Inflation Factor was also calculated, assuming no significant multi-collinearity when this value was <5. To investigate the incremental value of LARS over clinical and conventional echocardiographic parameters associated with the outcome, a likelihood ratio test was performed. The change in global χ^2^ values was calculated and reported. A two-tailed *P* value <0.05 was considered statistically significant. The inter- and intra-observer variability of LARS measurements were assessed by calculating the intra-class correlation coefficient on 20 randomly selected patients. The intra-class correlation coefficients for inter- and intra-observer variability were 0.92 (95% CI: 0.84–0.97, *P* < 0.001) and 0.94 (95% CI: 0.85–0.98, *P* < 0.001), respectively. Statistical analysis was performed using SPSS for Windows, version 25.0 (IBM Corporation, Armonk, NY, USA) and R version 4.0.1 (R Foundation for Statistical Computing, Vienna, Australia).

## Results

### Patient population

A total of 566 patients (age 64 ± 12 years; 66% men) was included from a cohort of 713 patients who underwent mitral valve repair for primary MR in our centre (Supplementary data online, *Figure S1*). Of note, patients who underwent mitral valve replacement (1.3%) were excluded, but because they met the other exclusion criteria, mentioned above. Baseline characteristics are shown in *Table 1*, while *Table 2* summarizes the echocardiographic data for the overall population. Of interest, LV function was in average preserved (LVEF was 64 ± 8%, LV GLS 21.4 ± 4.0%), systolic pulmonary arterial pressures were mostly within normal values [32 (25–42) mmHg], while LAVi was dilated in most patients with a median of 50 (39–67) mL/m^2^. Mean LARS was 23.0 ± 9.7%. Of interest, 406 (72%) patients underwent mitral valve surgery because of symptoms (of whom 70% were in NYHA II, 28% in NYHA III, and 2% in NYHA IV). Of the remaining 160 (28%) patients who were asymptomatic, 36 patients (6%) had signs of LV dysfunction (defined as LVEF ≤60% and/or LV end-systolic diameter ≥45 mm). Of the remaining 124 (22%) asymptomatic patients without LV dysfunction, 32 (6%) had an indication for surgery based on the presence of atrial fibrillation or pulmonary hypertension (>50 mmHg).

**Table 1:** Baseline clinical characteristics

	All patients (*n* = 566)	LARS ≤22% (*n* = 277)	LARS >22% (*n* = 289)	*P*-value
Age (years)	63.6 ± 12.3	67.3 ± 10.6	60.1 ± 12.9	**<0.001**
Male sex (%)	375 (66.3%)	176 (63.5%)	199 (68.9%)	0.184
Heart rate (bpm)	75 ± 20	80 ± 20	71 ± 18	**<0.001**
Systolic BP (mmHg)	135 ± 19	134 ± 20	136 ± 19	0.308
Diastolic BP (mmHg)	77 ± 11	77 ± 11	77 ± 11	0.961
BMI (kg/m^**2**^)	24.9 ± 3.4	25.1 ± 3.5	24.8 ± 3.4	0.348
Hypertension (%)	231 (40.8%)	112 (40.4%)	119 (41.2%)	0.865
Diabetes mellitus (%)	16 (2.8%)	8 (2.9%)	8 (2.8%)	1.000
Smoker (%)	186 (36.5%)	93 (37.5%)	93 (35.5%)	0.647
Coronary artery disease (%)	127 (23.1%)	71 (26.3%)	56 (20.0%)	0.086
COPD (%)	37 (6.7%)	23 (8.5%)	14 (5.0%)	0.125
eGFR (mL/min/1.73 m^**2**^)	79.9 ± 24.9	73.6 ± 23.1	86.0 ± 25.1	**<0.001**
CKD, eGFR < 60 mL/min/1.73 m^**2**^ (%)	116 (20.6%)	81 (29.2%)	35 (12.2%)	**<0.001**
Atrial fibrillation (%)	188 (33.2%)	144 (52.0%)	44 (15.2%)	**<0.001**
NYHA class ≥III (%)	120 (21.2%)	83 (30.0%)	37 (12.8%)	**<0.001**

Values in boldface are considered statistically significant (*p*-value <0.05). BMI, body mass index; BP, blood pressure; CKD, chronic kidney disease; COPD, chronic obstructive pulmonary disease; eGFR, estimated glomerular filtration rate; LARS, left atrial reservoir strain; NYHA, New York Heart Association.

**Table 2:** Baseline echocardiographic characteristics

	All patients (*n* = 566)	LARS ≤22% (*n* = 277)	LARS >22% (*n* = 289)	*P*-value
LVEDD (mm)	54.5 ± 6.5	54.6 ± 6.5	54.3 ± 6.5	0.563
LVESD (mm)	33.1 ± 6.8	34.0 ± 6.8	32.3 ± 6.7	**0.002**
LVEDV index (mL/m^2^)	71 ± 19	70 ± 21	72 ± 18	0.290
LVESV index (mL/m^2^)	24 (19–31)	24 (19–31)	24 (19–30)	0.415
LVEF (%)	64 ± 8	63 ± 9	66 ± 7	**<0.001**
LV GLS (%)	21.4 ± 4.0	19.9 ± 4.1	22.8 ± 3.3	**<0.001**
*E*/*e*′	12 (9–16)	13 (10–18)	11 (9–15)	**0.002**
sPAP (mmHg)	32 (25–42)	35 (29–48)	29 (25–35)	**<0.001**
EROA (cm^2^)	41 (29–54)	42 (31–55)	39 (29–53)	**0.048**
RVol (mL)	55 ± 23	57 ± 22	53 ± 24	0.064
Vena contracta (mm)	7.2 ± 1.7	7.5 ± 1.7	6.9 ± 1.7	**<0.001**
LAVi (mL/m^2^)	50 (39–67)	61 (45–84)	45 (35–54)	**<0.001**
LARS (%)	23.0 ± 9.7	15.3 ± 4.5	30.5 ± 6.9	**<0.001**

Values in boldface are considered statistically significant (*p*-value <0.05). EDD, end-diastolic diameter; EDV, end-diastolic volume; EF, ejection fraction; EROA, effective regurgitant orifice area; ESD, end-systolic diameter; ESV, end-systolic volume; GLS, global longitudinal strain; LARS, left atrial reservoir strain; LAVi, left atrial volume index; LV, left ventricular; RVol, regurgitant volume; sPAP, systolic pulmonary artery pressure.

### LARS strain and mortality after mitral valve surgery

After a median follow-up of 7 (4–12) years, 129 (22.8%) patients died. To investigate the association between LARS and all-cause mortality, spline curve analysis was performed and a LARS value of 22% was identified to dichotomize the population (i.e. in which the predicted HR was ≥1, *Figure 1*). Of note, this cut-off value was also close to the median value of the study population (22.3%). As shown in *Table 1*, patients with LARS ≤22% were significantly older, had more impaired renal function and were more symptomatic (NYHA functional class III to IV) compared with patients with LARS >22%. In terms of echocardiographic data (*Table 2*), patients with LARS ≤22% had a slightly larger LV end-systolic diameter; however, LV end-systolic volume indexed for body surface area was not significantly different among both groups. Furthermore, patients with LARS ≤22% had significantly lower LVEF and LV GLS and significantly higher systolic pulmonary artery pressures and LAVi compared with patients with LARS >22%.

**Figure 2:**
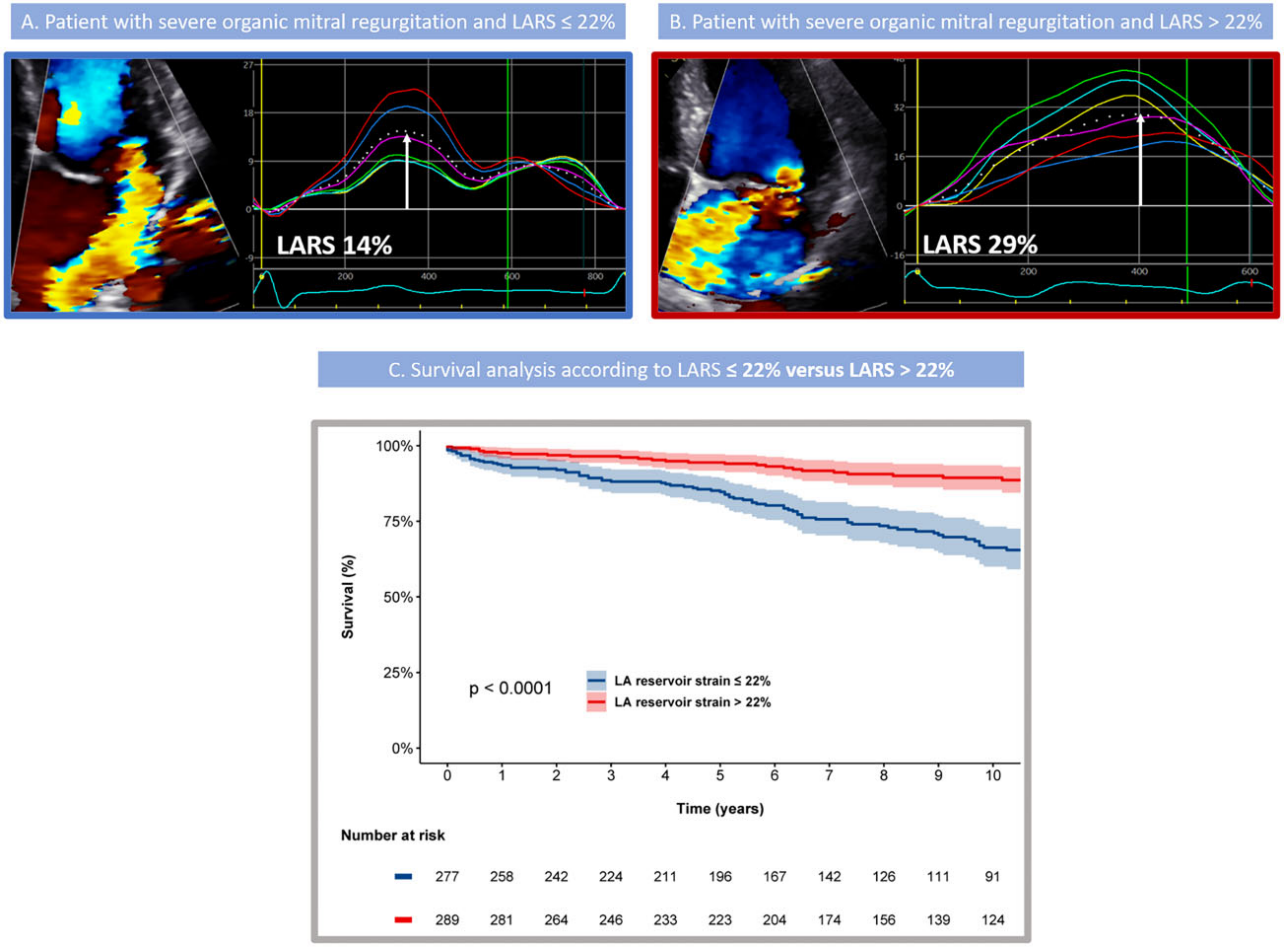
Association of LARS and all-cause mortality in patients with significant primary mitral regurgitation. Example of two patients having the same degree of mitral regurgitation and comparable LAVi, but different values for LARS: LARS 14% (A) and LARS 29% (B). LARS value is identified by the white arrows. Kaplan–Meier curves for all-cause mortality according to baseline LARS show that patients with LARS >22% have lower mortality rates compared with patients with LARS ≤22% (C). LARS, left atrial reservoir strain; LAVi, left atrial volume index.

**Figure 3:**
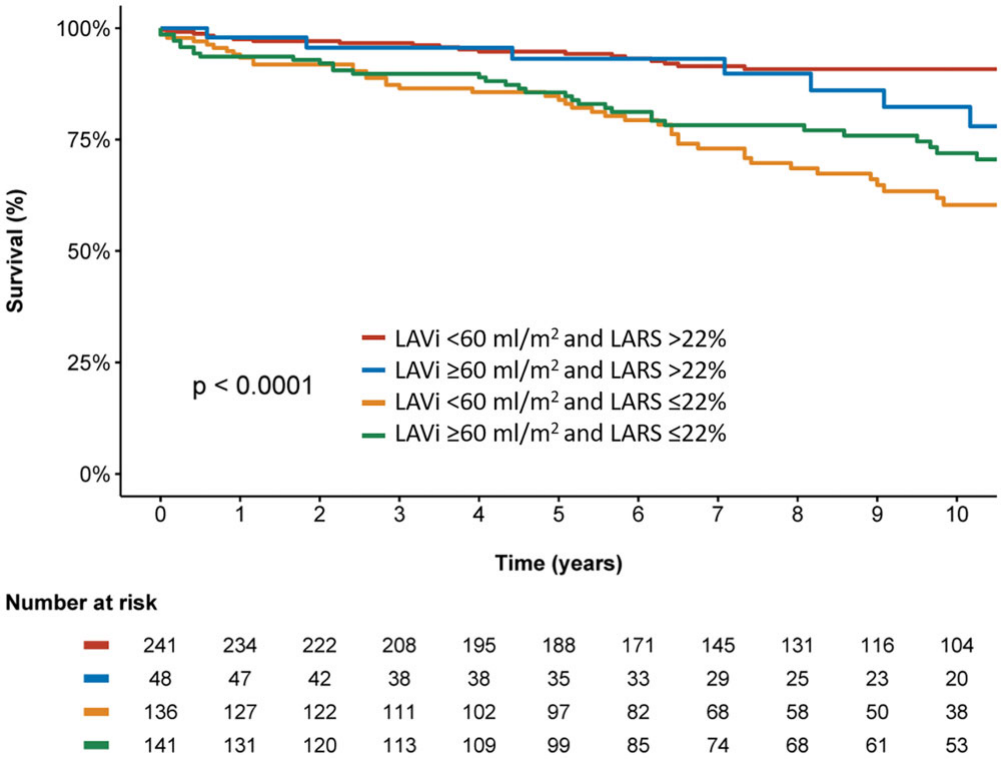
Kaplan–Meier curve for all-cause mortality according to LARS and LAVi. Time to all-cause mortality, according to baseline LARS and LAVi: LARS >22% and LAVi <60 mL/m2, LARS >22% and LAVi ≥60 mL/m2, LARS ≤22% and LAVi <60 mL/m2, and LARS ≤22% and LAVi ≥60 mL/m2. LARS, left atrial reservoir strain; LAVi, left atrial volume index.

During follow-up after surgery, patients with LARS ≤22% showed significantly higher mortality rates at 1-, 3-, and 5-year follow-up (6%, 12%, and 15%, respectively) when compared with patients with LARS >22% (2%, 3%, and 5%, respectively, log rank χ^2^ 35.1; *P* < 0.001, *Figure 2*). In addition, the Kaplan–Meier curve analysis was also performed dividing the population into four groups according to LARS and LAVi (*Figure 3*), demonstrating significantly higher cumulative mortality rates in patients with more pronounced LA adverse remodelling according to LARS (log rank χ^2^ 39.2; *P* < 0.001). Particularly for patients with LARS ≤22% (Groups 3 and 4), significantly higher event rates were noted compared with Group 1, independently of LA size (*P* < 0.001 for both). Furthermore, patients with LAVi ≥60 mL/m^2^ but LARS still >22% (Group 2) showed significantly lower mortality rates compared with patients with LAVi <60 mL/m^2^ but LARS ≤22% (Group 3, *P* = 0.018). In contrast, there was no significant difference between both groups with LARS >22% (Groups 1 and 2, *P* = 0.212) and both groups with LARS ≤22% (Groups 3 and 4, *P* = 0.141), demonstrating the incremental value of LARS over LAVi for risk assessment.

To investigate the association between LARS and all-cause mortality, a Cox proportional hazards model was constructed (*Table 3*). Univariable Cox hazard regression analysis showed that age, coronary artery disease, estimated glomerular filtration rate, NYHA functional class III–IV, previous atrial fibrillation, indexed LV end-diastolic volume, LVEF, LV GLS, LAVi, systolic pulmonary artery pressure, and LARS were associated with all-cause mortality. However, when including these variables into the multivariable analysis, only age, LV GLS, and LARS were independently associated with all-cause mortality (HR: 1.06; CI: 1.03–1.09; *P* < 0.001 for age; HR: 0.92; CI: 0.87–0.98; *P* = 0.014 for LV GLS; HR: 0.96; CI: 0.93–0.99; *P* = 0.014 for LARS).

**Table 3:** Univariable and multivariable Cox regression analyses

	Univariable analysis		Multivariable analysis	
	HR (95% CI)	*P*-value	HR (95% CI)	*P*-value
Age (years)	1.091 (1.068–1.114)	**<0.001**	1.061 (1.030–1.092)	**<0.001**
Male sex	0.876 (0.611–1.254)	0.468		
Coronary artery disease	1.680 (1.159–2.435)	**0.006**	0.944 (0.625–1.425)	0.782
eGFR (mL/min/1.73 m^2^)	0.967 (0.959–0.975)	**<0.001**	0.992 (0.981–1.004)	0.218
NYHA class ≥III	2.420 (1.692–3.462)	**<0.001**	1.477 (0.960–2.271)	0.076
Atrial fibrillation	2.068 (1.464–2.921)	**<0.001**	0.949 (0.618–1.459)	0.813
LVEDV index (mL/m^2^)	0.990 (0.981–1.000)	**0.044**	1.002 (0.991–1.013)	0.716
LVESV index (mL/m^2^)	1.002 (0.983–1.021)	0.868		
LVEF (%)	0.975 (0.956–0.996)	**0.017**	1.005 (0.977–1.034)	0.721
LV GLS (%)	0.881 (0.844–0.919)	**<0.001**	0.924 (0.868–0.984)	**0.014**
LAVi (mL/m^2^)	1.006 (1.002–1.011)	**0.009**	0.997 (0.989–1.005)	0.427
sPAP (mmHg)	1.017 (1.006–1.029)	**0.004**	0.997 (0.982–1.013)	0.753
EROA	1.005 (0.994–1.016)	0.349		
Rvol	1.008 (0.999–1.017)	0.101		
LARS (per % increase)	0.920 (0.899–0.941)	**<0.001**	0.961 (0.932–0.992)	**0.014**

Values in boldface are considered statistically significant (*p*-value <0.05). EDV, end-diastolic volume; EF, ejection fraction; eGFR, estimated glomerular filtration rate; ESV, end-systolic volume; EROA, effective regurgitation orifice area; GLS, global longitudinal strain; LARS, left atrial reservoir strain; LAVi, left atrial volume index; LV, left ventricular; NYHA, New York Heart Association; Rvol, regurgitant volume; sPAP, systolic pulmonary artery pressure.

#### Incremental prognostic value of LARS for all-cause mortality

**Figure 4:**
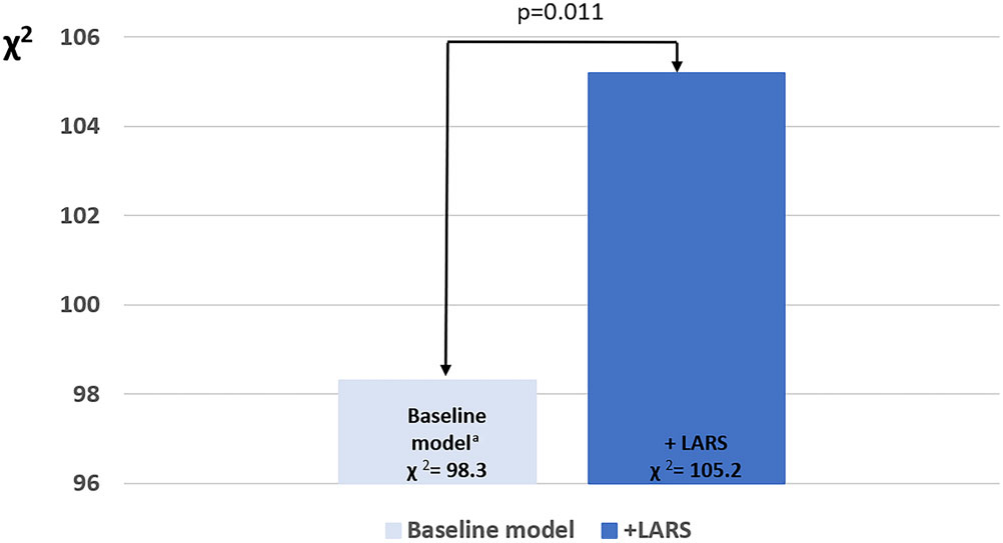
Likelihood ratio test for the incremental prognostic value of left atrial reservoir strain. The addition of LARS to a baseline clinical model is associated with significant increases in the χ^2^ value. ^a^The baseline model includes age, coronary artery disease, estimated glomerular filtration rate, NYHA III–IV, atrial fibrillation, left ventricular end-diastolic volume index, left ventricular ejection fraction, left ventricular global longitudinal strain, left atrial volume index, and systolic pulmonary artery pressure.

To determine the incremental prognostic value of LARS in addition to currently used clinical and conventional echocardiographic parameters, a likelihood ratio test was performed. The addition of LARS to a clinical model (including: age, coronary artery disease, estimated glomerular filtration rate, NYHA class III–IV, atrial fibrillation, LV end-diastolic volume index, LVEF, LV GLS, LAVi, and systolic pulmonary artery pressure) showed a significant increase in the χ^2^ value (χ^2^ difference = 6.9; *P* = 0.011), demonstrating the incremental prognostic value of LARS in patients with primary MR (*Figure 4*).

## Discussion

The main findings of the current study can be summarized as follows: (i) LARS is independently associated with all-cause mortality in patients undergoing mitral valve repair for severe primary MR; and (ii) LARS has incremental prognostic value over current clinical and echocardiographic risk factors for long-term survival.

### Prognostic implications of LA remodelling in primary MR

Primary MR induces LA volume and pressure overload, leading to progressive LA dilatation accompanied by an increase in interstitial fibrosis of the atrial wall and disarray of atrial muscle bundles.^23–25^ These structural changes subsequently cause a progressive reduction in atrial compliance with an increase in LA pressure, leading to important haemodynamic consequences. Since the LA functions as a reservoir between the left ventricle and the pulmonary vasculature, a reduced LA compliance in patients with MR increases the pulsatile loading on the pulmonary circulation, eventually leading to pulmonary hypertension and right ventricular—pulmonary arterial uncoupling.^26–30^ Furthermore, atrial remodelling also alters atrial electrical properties, thereby enhancing the risk of developing atrial fibrillation^31^ which has been associated with poor outcomes in patients with primary MR.^32–34^ Assessment of LA structural changes in patients with primary MR therefore seems essential to improve risk stratification and optimize timing of intervention. Previous studies have shown the relationship between LA size and outcome in patients with primary MR^9,10^ and current European guidelines suggest to consider mitral valve repair in the presence of significant LA dilatation (i.e. LAVi ≥60 mL/m^2^) or with new onset atrial fibrillation.^6^ However, chronic MR induces significant LA ultrastructural changes before LA dilatation occurs, thereby affecting LA myocardial contractility and relaxation at an earlier stage.^24,25,35^ Furthermore, LA dysfunction correlates better with LA compliance and may therefore represent an earlier stage of LA remodelling compared with LA dilation.^12,13^ In this regard, LARS measured by speckle tracking echocardiography has shown a good correlation with the extent of atrial fibrosis quantified by late gadolinium enhancement on cardiac magnetic resonance imaging^22^ and could therefore be used to improve risk stratification in patients with significant primary MR.

Initial studies in patients with primary MR have shown the association of LARS with the indication of mitral valve surgery. Ring *et al.*^15^ studied the clinical utility of LARS in 192 patients with mitral valve prolapse and different grades of MR severity (ranging from mild to severe), demonstrating the relationship between LA dysfunction and the presence of clinical indications for mitral valve surgery. Debonnaire *et al.*^8^ studied the prognostic value of LARS in 121 patients with severe MR, also showing that LARS was associated with the presence of conventional guideline-based mitral surgery indications. Although these findings suggest the value of a quantitative assessment of LA function to guide the optimal timing of surgery for primary MR, study populations were small and outcome data were lacking. The present study confirms the prognostic value of LARS in a large population including 566 patients with primary MR undergoing mitral valve repair and demonstrates the incremental prognostic value for all-cause mortality over standard measurements of LA size and LV size and function.

### Clinical implications

Appropriate timing for surgery and risk stratification in patients with severe primary MR remains challenging and therefore research has focused on identifying new and reliable prognostic parameters. The present study shows the prognostic value of LARS in patients with severe primary MR and specifically shows that patients with a more preserved LARS have significantly lower all-cause mortality. These findings could have two important implications. First, in asymptomatic patients with severe primary MR and without signs of LV remodelling, the presence of impaired LARS could help to select patients who may benefit from early surgery in highly experienced centres. Mitral valve surgery at this early stage might prevent patients from developing adverse LV remodelling, new-onset atrial fibrillation, and irreversible remodelling of the pulmonary vasculature. Second, in patients with LA dilatation, the presence of normal LARS could support the decision of watchful waiting in the absence of other criteria for intervention. However, randomized trials are needed to confirm these hypotheses.

### Limitations

This study is subject to the limitations of its retrospective, observational design. Because the study has been performed in a tertiary referral centre, highly experienced in mitral valve repair, the results from this cohort might not be generalizable to other centres. N-terminal pro-brain natriuretic peptide (NT-proBNP) was not systematically available and therefore could not be taken into account in the analysis. Exercise echocardiography was also not systematically performed in all asymptomatic patients or in patients with moderate to severe (instead of severe) MR, but the decision was left at the discretion of the treating physician. Furthermore, vendor-specific software was used, and this must be taken into consideration when assessing LARS with different software. Due to the limited number of asymptomatic patients without a classic indication for surgery, no definite statement can be made on the prognostic role of LARS in these patients. All-cause mortality was chosen as a primary endpoint as the exact cause of death could not be determined in all patients.

## Conclusions

LARS, a sensitive marker of LA function, is independently associated with all-cause mortality in patients with severe MR undergoing mitral valve repair. LARS may therefore be useful in the risk stratification of patients with primary MR and optimize timing of surgery.

## Supplementary Material

jeac058_Supplementary_Data

## Data Availability

The data underlying this article will be shared on reasonable request to the corresponding author.
