# Supplementary material for: Prognostic value of left atrial reservoir function in patients with severe primary mitral regurgitation undergoing mitral valve repair
Source: Eur Heart J Cardiovasc Imaging. 2022 Mar 18;24(1):142–51. doi: 10.1093/ehjci/jeac058 (PMC9762939; doi:10.1093/ehjci/jeac058)
Supplement: jeac058_Supplementary_Data [file jeac058_supplementary_data.docx]

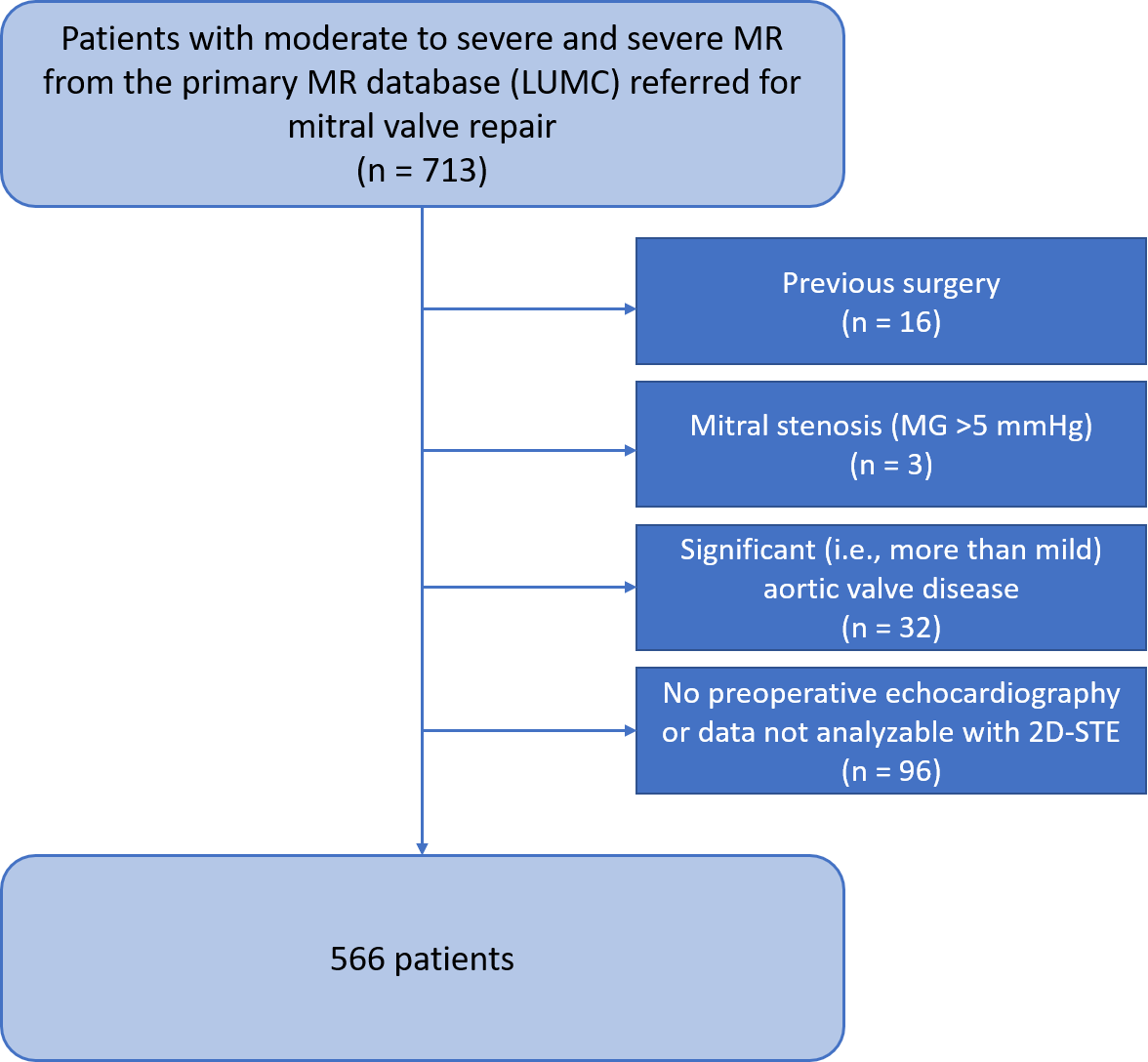
**Figure S1 – Study flow chart**

2D-STE = two dimensional – speckle tracking echocardiography; LUMC = Leiden University Medical Center; MG = mean gradient; MR = mitral regurgitation
